# Intersectional Racial and Sex Disparities in Unintentional Overdose Mortality

**DOI:** 10.1001/jamanetworkopen.2025.2728

**Published:** 2025-04-01

**Authors:** Kechna Cadet, Bianca D. Smith, Silvia S. Martins

**Affiliations:** 1 Department of Epidemiology, Columbia University Mailman School of Public Health, New York, New York; 2 Department of Mental Health, Johns Hopkins Bloomberg School of Public Health, Baltimore, Maryland

## Abstract

**Importance:**

There are several apparent research gaps based on the intersectional sociodemographic dimensions of drug-related mortality disparities. Relatively marginal evidence exists on the potential roles of intersecting forms of race and sexual marginalization on the disparities across drug-related mortality.

**Objective:**

To examine intersectional sex-specific White and Black racial disparities in drug poisoning mortality across states from 2010 to 2020.

**Design, Setting, and Participants:**

This cross-sectional study used unintentional fatal drug poisoning mortality data from the Centers for Disease Control and Prevention’s Web-Based Injury Statistics Query and Reporting System. Trends from 2010 through 2020 were analyzed by Black men, Black women, White men, and White women across states. One-sided Mann-Kendall trend analysis was used to examine statistically significant increasing or decreasing trends in age-adjusted mortality rates. Statistical analysis was performed from June to July 2024.

**Exposures:**

White and Black groups intersecting with male and female sex.

**Main Outcomes and Measures:**

Analysis of age-adjusted mortality rates, excess years of potential life lost (reference group: White men), years of potential life lost ratios (reference group: White men), and percentage change to highlight state differences.

**Results:**

From a total sample of 518 724 unintentional fatal drug poisoning deaths (9.0% in Black men, 3.9% in Black women, 29.0% in White women, and 58.1% in White men), 11 820 781 years of potential life lost were recorded. Black men had the highest mean (SD) age-adjusted mortality rate (23.25 [22.65]), followed by White men (22.49 [14.32]), with lower rates for White women (11.71 [5.96]) and Black women (9.01 [8.04]) (*P* < .001). Compared with White men, both Black men (τ = 0.298, slope = 0.002, intercept = 0.381, *P* < .001) and Black women (τ = 0.157, slope = 0.0004, intercept = 0.271, *P* < .001) had worsening YPLL over time, while White women (τ = -0.146, slope = −0.0003, intercept = 0.5252, *P* < .001) showed improvement in their YPLL over time compared with White men. At the state level, there was a disproportionate burden of deaths due to drug poisonings, with Maryland showing the highest increase in mortality rates among Black men (485.4%), while decreases were observed for White women in states such as Alaska (−23.0%).

**Conclusions and Relevance:**

In this cross-sectional study of overdose deaths, disparities in overdose mortality were evident, with Black men and Black women experiencing a pronounced and increasing burden of mortality compared with their White counterparts. Addressing these disparities will require a multipronged approach targeting the social, physical, economic, and policy risk environments.

## Key Points

**Question:**

Are there disparities by White and Black racial identity and sex in drug-related poisoning mortality across the US?

**Findings:**

In this cross-sectional study of 518 724 deaths from 2010 through 2020, Black men were particularly affected by drug-related poisoning mortality, as shown by the increasing trends in both age-adjusted mortality rates and years of potential life lost metrics relative to White men and Black and White women.

**Meaning:**

Interventions to improve drug-related poisoning mortality need to account for the intersectional identities and needs of specific communities, particularly Black men.

## Teaser

This cross-sectional study examines sex and racial disparities in drug-related deaths in the US.

## Introduction

Nearly 1 million people have died of a drug-related overdose since the opioid crisis began, making the opioid overdose epidemic one of the major contributors to the overall decline in life expectancy across the US,^1,2^ with trends following a nearly 25-year exponential increase in overdoses. Recently, national drug-involved overdose death data have shown that the current overdose epidemic is characterized by 4 salient waves that claimed a total 107 622 lives in 2021 alone.^3^ The drug market has evolved to include increasing opioid and stimulant polysubstance use (eg, fentanyl and methamphetamine).^1,4^ Racial disparity trends in the US overdose crisis have been highlighted in recent literature, with disproportionate rates observed among Black and American Indian or Alaska Native populations compared with White populations.^3,5,6,7,8^ By 2019, drug-related mortality for Black individuals exceeded that of their White counterparts in 23 US states, with the shift driven largely by higher rates of overdose due to fentanyl, heroin, and cocaine.^5^ However, little is known about the racial inequities in overdoses at the intersection of sex across states and time.

Historically, the overdose epidemic was dominated with the narratives surrounding the unequal burden of poisoning mortality rates on the White population, which was corroborated by the Centers for Disease Control and Prevention (CDC) Morbidity and Mortality Weekly Report, showing that, in 2016, 79% of all opioid-related deaths were among the White population (33 450 of 42 249 deaths).^9^ Data have shown that opioid and cocaine coinvolved overdose rates have plateaued among the non-Hispanic White population but increased among Black and Hispanic populations.^3^ Furthermore, the general national overdose rates have been higher across males compared to females, with the exceptions of a few states—namely, Idaho, Utah, and Arkansas, where heroin- and fentanyl-related deaths were higher among females in 2020.^10^ Epidemiologic research examining the distribution of drug-related mortality is often reported in additive terms, with comparisons made only by race and ethnicity, gender or sex, or age.^9,11,12^ This approach obscures the intersectional nature of the evolving epidemic and renders certain social strata invisible, with needs unmet.^11^

Intersectionality theory suggests that individuals are positioned within multidimensional interlocking structures of privilege and marginalization based on socially constructed identities of race, sex, and class.^13^ This framing views race and sex in a way that disrupts the tendency to view race and sex as separate structures, when they are inextricably intertwined.^13^ This paradigm is beneficial for parsing out the inequities associated with the pervasive drug-related mortality trends across the US. Although previous work has alluded to the shifting social profile of the US overdose crisis,^3^ less is known about the consequential disparities of marginalized race and sex identities, particularly for Black men and Black women. This shift may be driven in part by the changing drug market, with the increased fentanyl adulteration of local heroin, methamphetamine,^4^ and other drug supplies leading to unintentional polysubstance use. This study examines disparities and trends in drug poisoning mortality among Black men, Black women, White men, and White women from the 50 US states from 2010 through 2020. We aimed to identify intersectional sociodemographic subgroups and states that have experienced the highest rates of inequity and are in need of intervention.

## Methods

### Design and Database

Unintentional drug poisoning fatality data across all 50 states and the District of Columbia for the years 2010 through 2020 were obtained from the CDC Web-Based Injury Statistics Query and Reporting System (WISQARS) based on codes in the *International Statistical Classification of Diseases and Related Health Problems, Tenth Revision* (*ICD-10*). The number of deaths was determined from the annual mortality data file from the National Center for Health Statistics. Data include fatal injury data spanning across intent of injury (ie, unintentional injury, violence-related, homicide or assault, legal intervention, or suicide) and the mechanism by which a person sustained the fatal injury (ie, fall, fire, firearm, motor vehicle crash, poisoning, or suffocation). For this study, the dataset was filtered to include fatal unintentional poisoning fatalities across the 50 states and District of Columbia from January 1, 2010, to December 31, 2020. Drug poisoning (overdose) deaths were defined as having the underlying cause of death *ICD-10* code of X40-X44 (unintentional) and cause of injury by ingestion, inhalation, absorption through the skin, or injection of a drug, toxin, or chemical substance that resulted in a harmful outcome, such as a drug overdose. Types of drugs involved were indicated by the *ICD-10* multiple cause-of-death codes T40.1 (heroin), T40.2 (natural and semisynthetic opioids), T40.3 (methadone), T40.4 (synthetic opioids other than methadone), T40.5 (cocaine), and T43.6 (psychostimulants with abuse potential).^14^

For this cross-sectional study, we conducted a temporal analysis between January 1, 2010, and December 31, 2020, and a comparison of aggregate age-adjusted mortality rates per 100 000 population, mortality rate ratio and years of potential life lost (YPLL) ratio (with White men as the referent group), and percentage change in mortality rates from 2010 to 2014 and 2015 to 2020. eFigure 1 in Supplement 1 outlines how each metric was defined and calculated. The National Center for Health Statistics developed a race-bridging method in 2001 to allow comparison of race-specific trends over time.^15^ The bridged-race data (available for 2010-2020) were used for the temporal analysis of the race- and sex-specific temporal analyses and aggregated rate comparisons. The CDC censored small numbers of deaths to prevent patient identification; therefore, mortality data were unavailable for some strata. Data were analyzed from June to July 2024.

This study was determined exempt from approval and informed consent by the Columbia University Mailman School of Public Health Institutional Review Board due to the deidentified and publicly available nature of the data. This study followed the Strengthening the Reporting of Observational Studies in Epidemiology (STROBE) reporting guideline.

### Population and Exposures

Participants were adults aged 18 to 64 years from all 50 US states and Washington, DC, with race classified by the census bridged race and filtered to include the following intersectional race-sex groups: White men, White women, Black men, and Black women. White men were used as the referent group to highlight the disparities in marginalized racial and sex intersectional groups. The race and sex data are derived from WISQARS and are based on the classifications from death certificates.^14^ Other racial groups, including American Indian or Alaska Native populations, also experience high drug poisoning mortality rates, but limitations in the data availability and granularity across all states and years precluded their inclusion in this analysis.

### Statistical Analysis

#### Outcomes

Age-adjusted unintentional poisoning mortality rate–related (per 100 000 population) and YPLL-related metrics were compared from 2010 through 2020. Age-adjusted mortality rate is calculated by the CDC using the direct method and the 2000 US standard population and allows the ability to compare unintentional poisoning rates without being concerned that the differences in those rates are caused by variations in the age distributions between populations or among the same population over time.^16^ Similarly, YPLL is defined by the CDC as a measure of premature mortality (early death), which is calculated by subtracting the age at death from the standard year (ie, 65, 70, 75, 80, and ≥85) and then summing the individual YPLL across poisoning death.^17^ eFigure 1 in Supplement 1 lists the equations and definitions for metrics used in this study, and the eTable in Supplement 1 shows the descriptive statistics for the metrics from 2010 through 2020.

#### Temporal Analysis

The Mann-Kendall trend test is a nonparametric statistical test that is widely used to identify whether there is a significant change in the upward or downward trend in a set of time series data.^18,19^ The 2 advantages to using this test are that (1) it is a nonparametric test, and the data are not required to be normally distributed; and (2) it has low sensitivity to abrupt breaks in the time series due to inhomogeneity.^20^ According to this test, the null hypothesis (H0) assumes that the data are independent and randomly ordered (ie, there is no trend against the 1-sided alternative hypothesis), and the alternative hypothesis (H1) assumes that there is a trend that is either increasing or decreasing for each metric. This test was used to determine whether there are statistically significant increasing or decreasing trends in the age-adjusted mortality rate–related and YPLL-related metrics from 2010 to 2020 across the US based on the Kendall τ, which measures the strength, directionality, and magnitude based on the slope (β). A positive value of τ and β indicates an increasing trend, whereas negative values suggest a decreasing trend.

#### Statistical Software

Data management and statistical analysis were conducted in RStudio software, version 4.4.2 (R Project for Statistical Computing); specifically, the Mann-Kendall test was performed using the mk.test function from the trend package, version 1.1.6. The visualizations were created in Python, version 3.11.11 (Python Software Foundation) using the matplotlib, seaborn, and plotly libraries. One-sided *P* < .05 indicated statistical significance.

## Results

### Descriptive Analysis

The final analytic sample consists of 518 724 unintentional fatal drug poisoning deaths, distributed as follows: 46 776 Black men (9.0%), 20 087 Black women (3.9%), 150 405 White women (29.0%), and 301 456 White men (58.1%), resulting in 11 820 781 YPLL from 2010 to 2020. A summary of unintentional fatal drug poisoning mortality across the study population, stratified by intersectional Black and White race and sex groups, is shown in Table 1. There were significant differences in the mean (SD) number of deaths between groups (48.75 [53.67] in Black women, 105.59 [133.80] in Black men, 275.97 [282.32] in White women, and 543.16 [645.93] in White men; *P* < .001). The mean (SD) age-adjusted mortality rate was highest among Black men (23.25 [22.65]), which was slightly higher than the rate in White men (22.49 [14.32]). White women and Black women had lower mean (SD) rates at 11.71 (5.96) and 9.01 (8.04), respectively. Differences in these age-adjusted mortality rates across groups were statistically significant (*P* < .001).

**Table 1.** Summary of Unintentional Fatal Drug Poisoning Mortality Stratified by Intersectional Race and Sex in the United States, 2010-2020

Variable	Black		White		*P* value
	Women	Men	Women	Men	
Deaths, mean (SD)	48.75 (53.67)	105.59 (133.80)	275.97 (282.32)	543.16 (645.93)	<.001
Age-adjusted mortality rate, mean (SD)	9.01 (8.04)	23.25 (22.65)	11.71 (5.96)	22.49 (14.32)	<.001
Excess YPLL, mean (SD)	−329.70 (142.94)	−57.78 (357.82)	−285.24 (159.34)	1 [Reference]	<.001

Abbreviation: YPLL, years of potential life lost.

Compared with White men (referent group), Black men (mean [SD], −57.78 [357.82]), White women (mean [SD], −285.24 [159.34]), and Black women (mean [SD], −329.70 [142.94]) had significantly fewer YPLL, respectively. Differences observed in excess YPLL across groups were statistically significant (*P* < .001).

**Figure 1.**
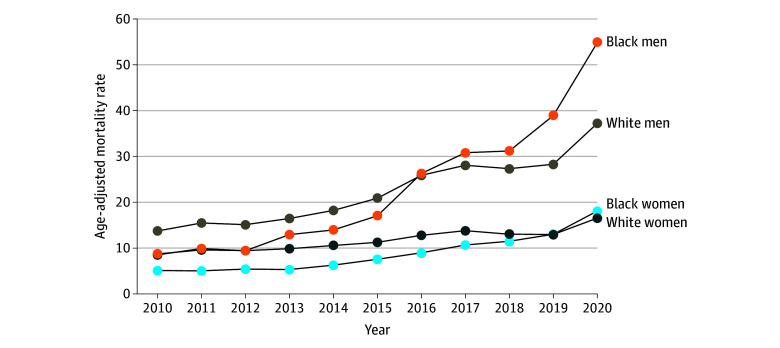
Trends in Age-Adjusted Unintentional Drug Poisoning Mortality Rates Across Intersectional Race and Sex, 2010 to 2020

Figure 1 shows the Black-White age-adjusted mortality rate for men and women. It illustrates how the mortality rate for all intersectional Black and White race-sex groups increased from 2010 to 2020. The mortality rate for Black men surpassed the rate for their White male counterparts in 2016 and was nearly 60% higher than for White men by 2020 (eFigure 2 in Supplement 1). Similarly, there was a gradual increase in the mortality rate for Black women, which surpassed that of their White female counterparts in 2019. This finding suggests that Black women are now experiencing slightly higher rates than White women.

### Temporal Analysis of Age-Adjusted Unintentional Drug Poisoning Mortality Rate and YPLL Ratio Across Intersectional Racial and Sex Populations

#### Trends in Excess YPLL

There were significant temporal trends in excess YPLL in unintentional drug poisoning across the intersectional race and sex groups. A decreasing trend in excess YPLL was observed for White women (τ = −0.463, β = −0.657, intercept = −77.140, *P* < .001), and Black women (τ = −0.331, β = −0.699, intercept = −195.214, *P* < .001). However, Black men experienced an increasing trend in excess YPLL (τ = 0.141, β = 0.443, intercept = −231.682, *P* < .001).

#### Trends in YPLL Ratio

Using White men as the reference group for the YPLL ratio, we observed significant increasing temporal trends among Black men (τ = 0.298, β = 0.002, intercept = 0.381, *P* < .001) and Black women (τ = 0.157, β = 0.0004, intercept = 0.27, *P* < .001), which indicate worsening YPLL ratios relative to their White male counterparts. Conversely, White women showed a significant decreasing YPLL ratio temporal trend (τ = −0.146, β = −0.0003, intercept = 0.525, *P* < .001), suggesting an improvement in their YPLL ratio over time.

#### Trends in Age-Adjusted Mortality Rates

As shown in Table 2, there were increasing trends in age-adjusted unintentional drug poisoning mortality rates for Black men (τ = 0.488, β = 0.085, intercept = 1.418, *P* < .001), White men (τ = 0.323, β = 0.032, intercept = 9.323, *P* < .001), and Black women (τ = 0.366, β = 0.034, intercept = 2.995, *P* < .001), although less pronounced. White women had the least pronounced increasing trend (τ = 0.227, β = 0.011, intercept = 7.580, *P* < .001).

**Table 2.** Mann-Kendall Trend Test Results

Metric	τ^a^	β^b^	Intercept^c^	*P* value	Trend description^d^
**Black women**					
Excess YPLL	−0.331	−0.699	−195.214	<.001	Decreasing
YPLL ratio	0.157	0.0004	0.271	<.001	Increasing
Age-adjusted mortality rate	0.366	0.034	2.995	<.001	Increasing
**Black men**					
Excess YPLL	0.141	0.443	−231.682	<.001	Increasing
YPLL ratio	0.298	0.002	0.381	<.001	Increasing
Age-adjusted mortality rate	0.488	0.085	1.418	<.001	Increasing
**White women**					
Excess YPLL	−0.463	−0.657	−77.140	<.001	Decreasing
YPLL ratio	−0.146	−0.0003	0.525	<.001	Decreasing
Age-adjusted mortality rate	0.227	0.011	7.580	<.001	Increasing
**White men**					
Excess YPLL	1 [Reference]	NA	NA	NA	NA
YPLL ratio	1 [Reference]	NA	NA	NA	NA
Age-adjusted mortality rate	0.323	0.032	9.323	<.001	Increasing

Abbreviations: NA, not applicable; YPLL, years of potential life lost.

^a^ Kendall τ coefficient indicating the direction and strength of the trends, with positive values indicating increasing trends and negative values indicating decreasing trends.

^b^ β quantifies the magnitude of change in the metrics per unit increase in year.

^c^ Intercept represents the estimated value of the metric at the starting point.

^d^ Trend description describes whether the trend from 2010 to 2020 is increasing, decreasing, or not significant based on the *P* value.

### Intersectional Racial and Sex Disparities in YPLL in the US, 2010-2020

**Figure 2.**
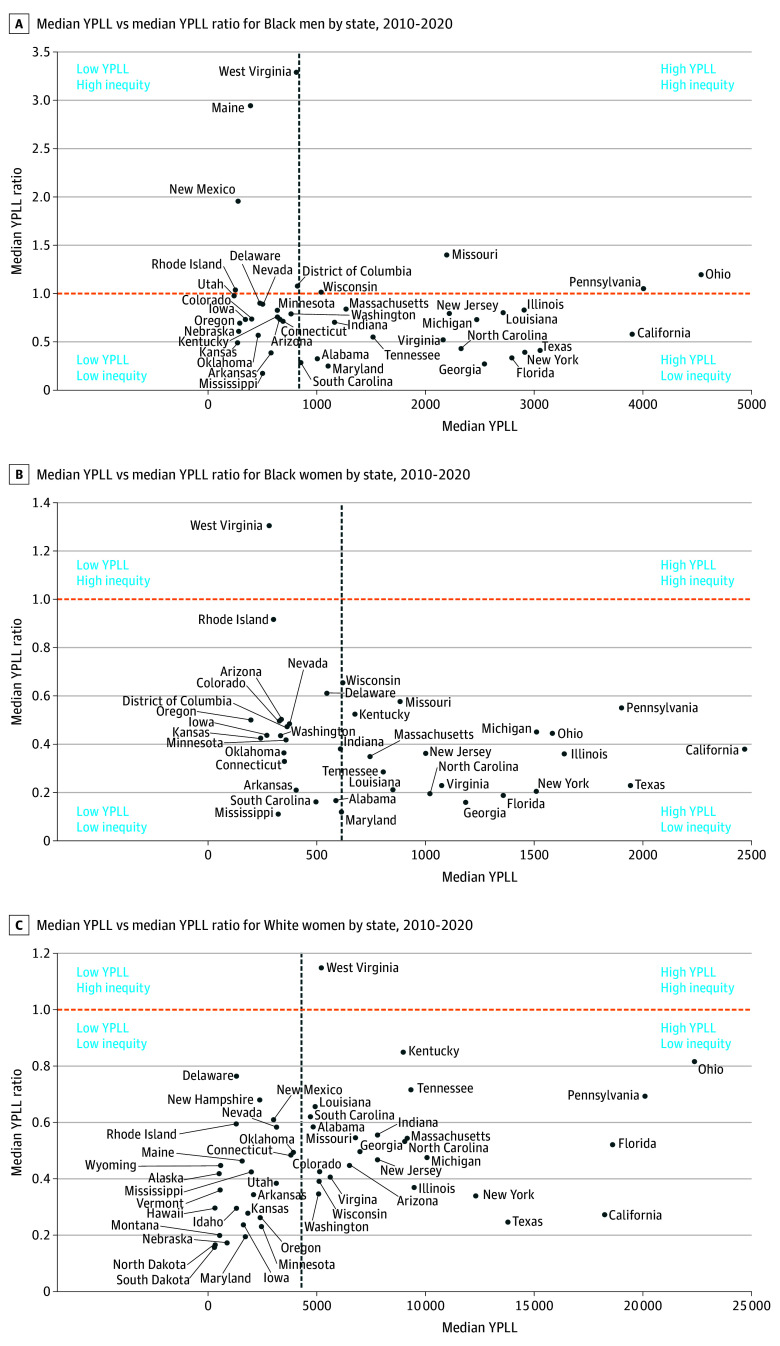
Comparison of Years of Potential Life Lost (YPLL) and Inequities Between Intersectional Black and White Male and Female Populations Across the 50 US States and District of Columbia A, Nine states do not appear in the graph due to missing median YPLL for Black men during the 2010-2020 period: Alaska, Hawaii, Idaho, Montana, New Hampshire, North Dakota, South Dakota, Vermont, and Wyoming. This indicates that there were either no reported drug poisoning deaths among Black men in these states or that YPLL data were insufficient for median calculations. B, Thirteen states do not appear in the graph due to missing median YPLL for Black women during the 2010-2020 period: Alaska, Hawaii, Idaho, Maine, Montana, Nebraska, New Hampshire, New Mexico, North Dakota, South Dakota, Utah, Vermont, and Wyoming. This indicates that there were either no reported drug poisoning deaths among Black women in these states or that YPLL data were insufficient for median calculations. C, The District of Columbia does not appear in the graph due to missing YPLL data for White women during the 2010-2020 period. This indicates that there were either no reported drug poisoning deaths among White women in the District of Columbia or that YPLL data were insufficient for analysis. The horizontal dashed lines indicate YPLL rate ratios; vertical dashed lines, US median YPLL between Black and White individuals across sex.

We plotted the 50 states and District of Columbia according to their median YPLL vs the median YPLL ratio for intersectional race and sex across various states from 2010 to 2020 (Figure 2). For Black men, 4 states were classified as worst performing, including Ohio, Pennsylvania, Missouri, and Wisconsin. Additionally, there were 17 states (including California, Texas, Illinois, New York, Florida, Georgia, Louisiana, New Jersey, Virginia, and Maryland) where Black men experienced higher than the median YPLL compared with the rest of the US, although Black men residing in these states did not experience greater median YPLL than their White counterparts. White women experienced higher YPLL and sex-related disparity in West Virginia compared with their White male counterparts. There were 24 states where White women experienced lower racial disparities but higher than the median YPLL compared with the US overall. There were 19 states where Black women experienced higher median YPLL but lower racial disparities than the country as a whole.

### Percentage Change in Mean Age-Adjusted Unintentional Drug Poisoning Mortality Rates by Intersectional Racial and Sex Populations: 2010-2014 vs 2015-2020

**Figure 3.**
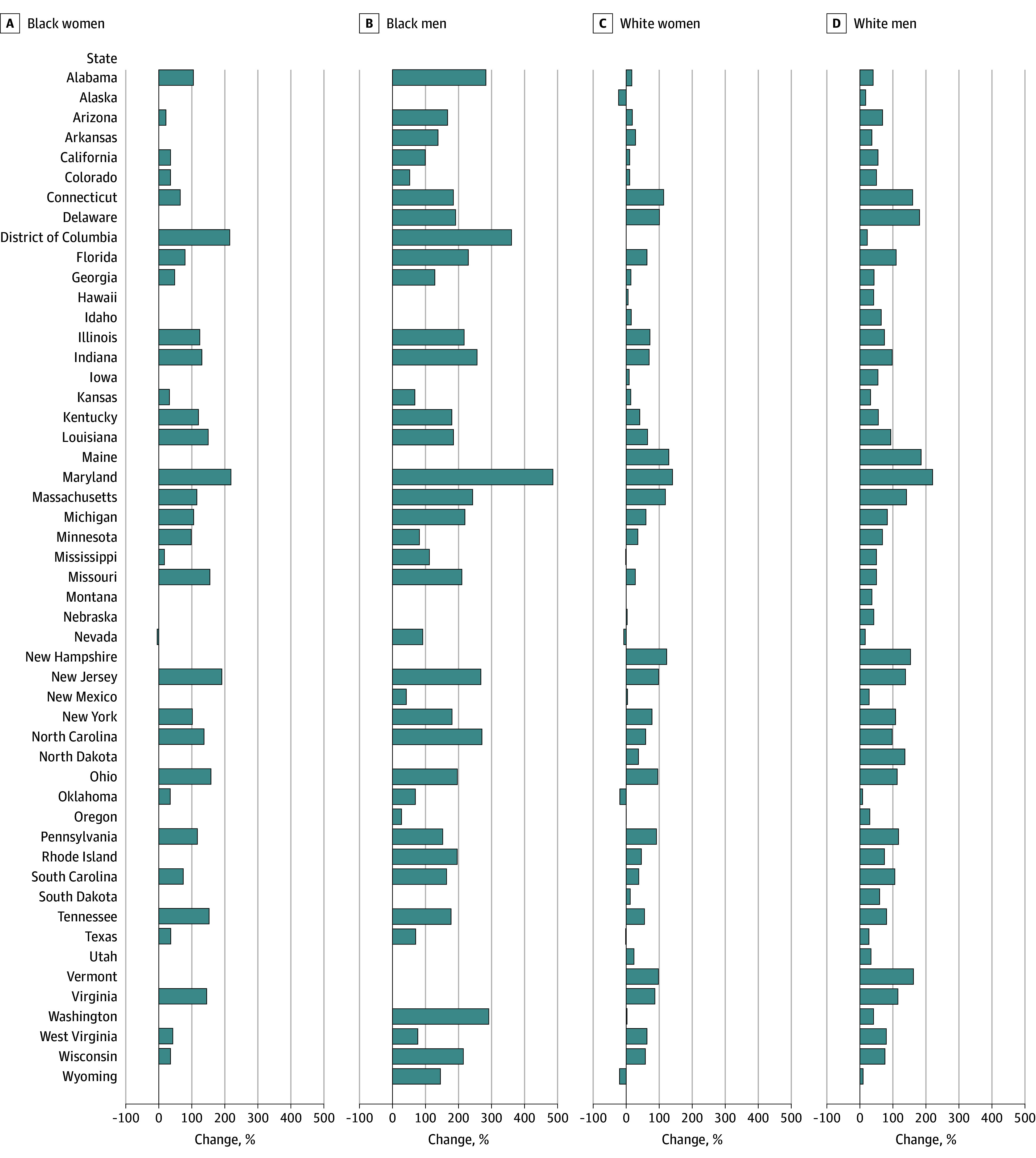
Percentage Change in Age-Adjusted Mortality Rate for Unintentional Fatal Drug Poisoning Stratified by Intersectional Race and Sex, 2010-2014 vs 2015-2020

We explored the change in age-adjusted unintentional drug poisoning mortality rate, comparing the 2010 to 2014 period with the 2015 to 2020 period based on the third and fourth waves of the overdose epidemic, representing the fentanyl and polysubstance use waves, respectively. Figure 3 presents the percentage change stratified by the intersectional race and sex groups. States with the most substantial change for Black men included Maryland, with a 485.4% increase in unintentional drug poisoning fatality rate in 2015 to 2020, followed by the District of Columbia (360.4%), Virginia (291.1%), Alabama (282.8%), North Carolina (270.9%), and New Jersey (267.5%). Contrastingly, Alaska, Oklahoma, and Wyoming experienced a decrease in White female drug poisoning mortality, reporting 23.0%, 19.1%, and 20.1% decreases, respectively (Figure 3).

## Discussion

In this cross-sectional study, we examined intersectional sex-specific White and Black racial disparities in drug poisoning mortality across states from 2010 through 2020. The results indicate significant disparities in drug poisoning mortality and life loss metrics at the intersection of race and sex demographic groups. The age-adjusted drug poisoning mortality rate for Black men surpassed that of their White male counterparts in 2016, and the rate for Black women surpassed that of their White female counterparts in 2019. The magnitude and direction of the temporal trend of YPLL ratio were more pronounced for Black men and Black women and are increasing, denoting a disproportionate burden of deaths due to drug poisonings, especially in Ohio, Pennsylvania, Missouri, and Wisconsin for Black men. Findings further emphasize the importance of future studies to intentionally examine intersectional groups within the context of the drug overdose landscape.

Maryland had the highest percentage change for Black men, experiencing a 485.4% increase in unintentional drug poisoning mortality among Black men between 2015 and 2020, which coincides with the fourth wave of the overdose epidemic. Previous studies have suggested an increasingly toxic drug supply, characterized by unintentional polysubstance use combinations of fentanyl, stimulants, and benzodiazepines, which is contributing to the worsening drug poisoning mortality rates across the nation.^21,22^ The unpredictability of the new drug supply landscape may be disproportionately impacting Black men due to structural determinants such as unstable housing, unemployment, inequitable access to harm reduction services and medications for opioid use disorder, and exposure to excessive policing and trauma, to name a few.^23,24^

Effective measures are required to mitigate the increasing trend in mortality rates and YPLL among Black men and Black women. The underlying mechanisms explaining these epidemiologic findings cannot be directly determined from these data, but sex differences in the propensity to develop substance use disorder^25^ and sex-specific biological vulnerability to drug toxicity^26^ are not sufficient to explain the intersectional race and sex differences in mortality rates. Although this study did not examine social (eg, poverty) and structural (eg, racism) factors among decedents, other studies report persistent structural racism that permeates the US drug environment.^27,28^ Therefore, drawing on evidence-based public health research and practice is necessary to curb the worsening and disproportionate deaths that are affecting Black people. Harm reduction services and evidence-based treatment programs have been lacking among predominantly Black neighborhoods^27^; therefore, state and local funding is needed to implement these programs in these geographic regions. Additionally, overdose prevention centers have a high degree of acceptability and willingness to use among White and Black populations; therefore, these centers could be an additional resource across states with high mortality rates.^29^ One notable challenge is community stigma toward individuals who use drugs, often manifesting as “not in my backyard” attitudes, with concerns that harm reduction services may attract undesirable or dangerous individuals to local communities.^30^ These perceptions can hinder the expansion of essential harm reduction programs.^30^ Future research should explore how targeted informational messaging can be used to build community support for these efforts. By fostering greater understanding and reducing stigma, these efforts can play a critical role in addressing the ongoing national overdose crisis, especially at the intersection of race and sex.

### Limitations

This study has some limitations. Our estimates do not delineate between Hispanic and non-Hispanic Black and White populations; therefore, we recognize that our disparity analyses are limited to examining differences without accounting for the additional intersectionality of ethnicity. We examined disparities related to male and female sex recorded on the death records, not self-reported gender; therefore, our analyses do not consider the unique intersectional disparities relating to disproportioned mortality and YPLL for sexual and gender minority populations. Furthermore, drug poisoning deaths may be misclassified as undetermined across many states^31^ due to the challenge of determining the intent of overdose.^32^ This misclassification could result in an underreporting of unintentional drug poisoning deaths; therefore, the percentage change in mortality may be higher than what is reported in this study.

## Conclusions

In this cross-sectional study, disparities in fatal drug poisoning mortality were evident, with Black men and Black women experiencing a pronounced and increasing burden of mortality compared with their White counterparts. Our decomposition of intersectional race and sex disparities relating to drug poisoning mortality and YPLL during a decade provides valuable information that can be used to target populations that require the highest attention by state stakeholders, health departments, and advocacy partners.

## Notes

Supplement 1. **eTable.** Trends in Unintentional Fatal Drug Poisoning Mortality Disparities by Intersectional Race and Sex Across the United States From 2010-2020 **eFigure 1.** Equations and Definitions for the Metrics Used in This Study **eFigure 2**. Trends in Age-Adjusted Unintentional Drug Poisoning Mortality Rate Ratio Across Intersectional Race and Sex, 2010-2020

Supplement 2.
Data Sharing Statement
