# Supplementary material for: Intersectional Racial and Sex Disparities in Unintentional Overdose Mortality
Source: JAMA Netw Open. 2025 Apr 1;8(4):e252728. doi: 10.1001/jamanetworkopen.2025.2728 (PMC11962664; doi:10.1001/jamanetworkopen.2025.2728)
Supplement: Supplement 1. — eTable. Trends in Unintentional Fatal Drug Poisoning Mortality Disparities by Intersectional Race and Sex Across the United States From 2010-2020 eFigure 1. Equations and Definitions for the Metrics Used in This Study eFigure 2. Trends in Age-Adjusted Unintentional Drug Poisoning Mortality Rate Ratio Across Intersectional Race and Sex, 2010-2020 [file jamanetwopen-e252728-s001.pdf]

Supplemental Online Content

Cadet K, Smith BD, Martins SS. Intersectional racial and sex disparities in unintentional overdose mortality. *JAMA Netw Open*. 2025;8(4):e252728. doi:10.1001/jamanetworkopen.2025.2728

eTable. Trends in Unintentional Fatal Drug Poisoning Mortality Disparities by Intersectional Race and Sex Across The United States From 2010-2020

eFigure 1. Equations and Definitions for the Metrics Used in This Study

eFigure 2. Trends in Age-Adjusted Unintentional Drug Poisoning Mortality Rate Ratio Across Intersectional Race and Sex, 2010-2020

This supplemental material has been provided by the authors to give readers additional information about their work.

© 2025 Cadet K et al. *JAMA Network Open*.

eTable. Trends in Unintentional Fatal Drug Poisoning Mortality Disparities by Intersectional Race and Sex Across The United States From 2010-2020

	2010-2014				2015-2020				Total			
	Excess YPLL	YPLL Ratio	Age-adjusted Mortality Rates	Mortality rate ratio	Excess YPLL	YPLL Ratio	Age-adjusted Mortality Rates	Mortality rate ratio	Excess YPLL	YPLL Ratio	Age-adjusted Mortality Rates	Mortality rate ratio
**Race**												
**White** Mean (SD)	-90.6 (159.5)	0.76 (0.42)	12.7 (6.6)	0.57 (0.29)	-182.7 (327.7)	0.72 (0.50)	20.7 (14.4)	0.92 (0.64)	-141.3 (269.5)	0.74 (0.46)	17.1 (12.2)	0.76 (0.54)
**Black** Mean (SD)	-196.4 (130.2)	0.49 (0.34)	8.3 (7.3)	0.37 (0.32)	-174.0 (391.24)	0.71 (0.59)	22.8 (22.1)	1.01 (0.98)	-183.3 (310.9)	0.62 (0.51)	16.4 (18.6)	0.73 (0.83)
**Intersectional Race-Sex Identity**												
**White-Male** Mean (SD)	0.00 (163.6)	1.00 (0.43)	15.8 (7.2)	0.70 (0.32)	0.00 (348.2)	1.00 (0.53)	27.9 (16.2)	1.24 (0.72)	0.00 (280.6)	1.00 (0.49)	22.5 (14.3)	1.00 (0.64)
**Black-Male** Mean (SD)	-146 (145.6)	0.62 (0.38)	10.9 (8.3)	0.48 (0.37)	6.7 (442.8)	0.98 (0.66)	33.4 (25.5)	1.49 (1.1)	-57.8 (357.8)	0.83 (0.59)	23.2 (22.6)	1.03 (1.0)
**White-Female** Mean (SD)	- 182.6(85.5)	0.52 (0.22)	9.65 (4.0)	0.43 (0.18)	-369 (156.7)	0.43 (0.22)	13.4 (6.7)	0.60 (0.30)	-285.2 (159.3)	0.47 (0.22)	11.7 (6.0)	0.52 (0.26)
**Black-Female** Mean (SD)	-257 (72.0)	0.33 (0.18)	5.45 (4.5)	0.24 (0.20)	-379.8 (157.6)	0.41 (0.25)	11.7 (9.0)	0.52 (0.40)	-329.7 (142.9)	0.38 (0.22)	9.0 (8.0)	0.40 (0.36)
Notes: This data included 518,724 unintentional fatal drug poisoning deaths across the US including Hawaii and Alaska.												

© 2025 Cadet K et al. *JAMA Network Open*.

eFigure 1. Equations and Definitions for the Metrics Used in This Study

◊ **Age-adjusted mortality rate ratios (AAMRR).** The ratio of age-adjusted mortality rates for each race-sex groups (Black females, White females, White males) to that of White males

$$\text{AAMRR}=\frac{Age-Adjusted\ Mortality\ Rate\ of\ Group\ A}{Age-Adjusted\ Mortality\ Rate\ of\ Group\ B}$$

◊ **Percent Change in mortality rate from 2010-2014 and 2015-2020.**

$$\text{Percent Change}=\frac{(Mortality\ Rate\ from\ 2015-2020)-(Mortality\ Rate\ from\ 2010-2014)}{Mortality\ Rate\ from\ 2010-2014}$$

◊ **Years of Potential Life Lost (YPLL) rate ratios.** YPLL rate ratios is used to compare the rate of YPLL per 100,000 population of each race-sex groups (Black females, White females, White males) to that of White males.

$$\text{YPLL Rate Ratio}=\frac{YPLL\ Rate\ per\ 100{,}000\ of\ Group\ A}{YPLL\ Rate\ per\ 100{,}000\ of\ Group\ B}$$

© 2025 Cadet K et al. *JAMA Network Open*.

eFigure 2. Trends in Age-Adjusted Unintentional Drug Poisoning Mortality Rate Ratio Across Intersectional Race and Sex, 2010-2020

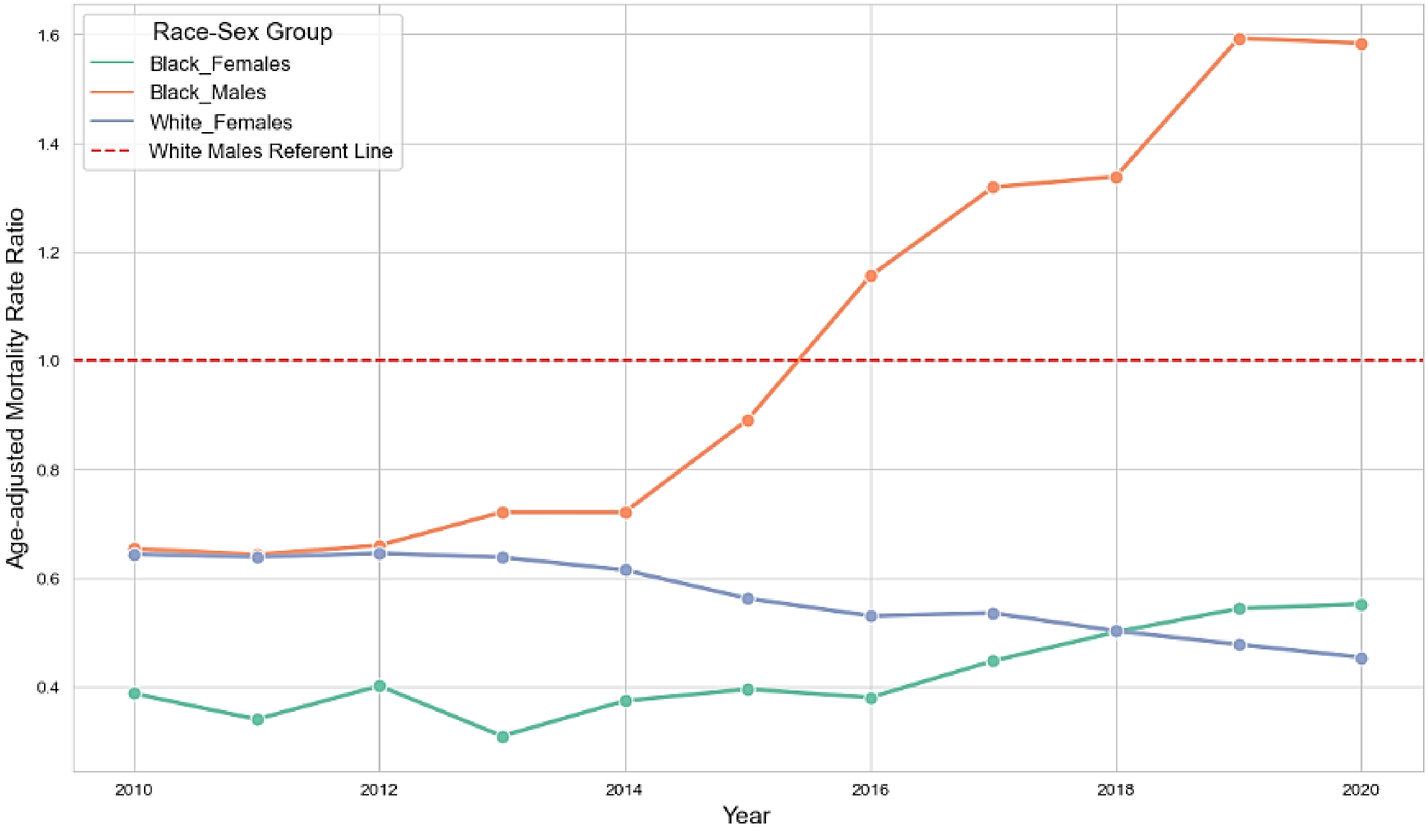

eFigure 2 shows mortality rate ratios for intersectional race-sex groups for White males, White females, Black males, Black females. The mortality rate ratio for Black males began at 0.70 in 2010 and grew gradually over the next ten years, eventually surpassing the 1.00 mark between 2015 and 2016. By 2020, the mortality rate for Black males were nearly 60% higher than that of White males. Furthermore, mortality rates of White and Black females are lower than those of White males, with White females experiencing decreasing mortality rates from 2012 to 2020 compared to their White male counterparts.

© 2025 Cadet K et al. *JAMA Network Open*.
