# Supplementary material for: Intersectional Racial and Sex Disparities in Unintentional Overdose Mortality
Source: JAMA Netw Open. 2025 Apr 1;8(4):e252728. doi: 10.1001/jamanetworkopen.2025.2728 (PMC11962664; doi:10.1001/jamanetworkopen.2025.2728)
Supplement: Supplement 2. — Data Sharing Statement [file jamanetwopen-e252728-s002.pdf]

Data Sharing Statement

Cadet. Intersectional Racial and Sex Disparities in Unintentional Overdose Mortality. *JAMA Netw Open*. Published April 01, 2025. doi:10.1001/jamanetworkopen.2025.2728

Data
Data available: Yes
Data types: Deidentified participant data
How to access data: https://wisqars.cdc.gov
When available: With publication

Supporting Documents
Document types: Statistical/analytic code
How to access documents: The statistical/analytic code can be made available upon request. Please email kc3010@cumc.columbia.edu.
When available: With publication

Additional Information
Who can access the data: Anyone requesting the data
Types of analyses: The data is publicly available data and can be accessed and used per CDC regulations.
Mechanisms of data availability: The data is publicly available data and can be accessed and used per CDC regulations.
